# An ARFID case report assessment with 18F-FDG PET/MRI and treatment with olanzapine, escitalopram and fluoxetine in adult

**DOI:** 10.3389/fpsyt.2024.1450800

**Published:** 2025-01-15

**Authors:** Meichen Liu, Yiheng Chang, Xueting Xie, Yudan Liu, Shiyun Tian, Chun Yang, Ziqi Zhao, Huimin Zhang

**Affiliations:** ^1^ Department of Neurology, the First Affiliated Hospital, Dalian Medical University, Dalian, China; ^2^ Department of Radiology, the First Affiliated Hospital, Dalian Medical University, Dalian, China; ^3^ Department of General Surgery, the First Affiliated Hospital, Dalian Medical University, Dalian, China

**Keywords:** avoidant/restrictive food intake disorder, magnetic resonance imaging, positron emission tomography, olanzapine, escitalopram, fluoxetine, neuroimaging, case report

## Abstract

**Background:**

Avoidant/restrictive food intake disorder (ARFID), a DSM-5-introduced eating disorder, is increasingly prevalent and challenging to treat, primarily affecting children and adolescents, with limited adult case reports. This rarity in adults leads to misdiagnosis or underdiagnosis, and treatment experiences are scarce.

**Case presentation:**

This report details an adult ARFID case, where the patient’s fear of food intake followed gastric damage from corn ingestion, resulting in a restrictive diet of rice porridge due to gastric pain. The behavior is driven by fear of eating-related adverse effects.

**Result:**

Based on clinical symptoms assessment, semi-structured interviews, and comprehensive testing, including PET/MRI scans to exclude secondary conditions, a diagnosis of ARFID was confirmed. ARFID, a newly introduced diagnosis in the DSM-5, is uncommon in adults with sparse case data. Differentiating it from gastrointestinal diseases and other psychiatric conditions is crucial for precise diagnosis and focused treatment.

**Conclusion:**

In this paper, we report on the clinical diagnosis, imaging manifestations, treatment, and follow-up of an adult ARFID case, highlighting the utility of neuroimaging in diagnosis and differentiation. It also assesses the therapeutic efficacy of olanzapine, escitalopram oxalate, and fluoxetine hydrobromide, offering clinical guidance for diagnosing and managing ARFID.

## 1 Introduction

Avoidant/restrictive food intake disorder (ARFID) refers to a condition in which patients reduce the variety and/or total amount of food intake due to a lack of interest in eating. As a unique diagnostic category, it has been newly included in the DSM-5 as an eating disorder disease (1). It typically occurs in infancy or early childhood but can also happen at any age. It is more harmful and may lead to nutrition disorders if eating is not adjusted in a timely manner. Recently, there has been a proposal that for these patients who do not fit the ‘typical’ criteria for eating disorders, there is still a lack of consensus on the best diagnostic and treatment methods. Currently, three distinct clinical manifestations are more widely recognized: reduced sensory sensitivity, fear of negative consequences, and a lack of interest in eating (2). Additionally, some studies have established a three-dimensional clinical diagnostic model based on this, which includes a lack of interest in food and eating, avoidance of food due to discomfort based on sensory sensitivity, and fear of aversive outcomes due to previous or anticipated negative experiences (3). It is important to note that a key difference between ARFID and other restrictive eating disorders (such as anorexia nervosa) is that patients with ARFID do not have a fear of weight gain and do not engage in behaviors that interfere with weight or shape (4).

It has been reported that ARFID is mainly related to the following factors, such as age and gender, with some studies suggesting that males are more prone to ARFID (5). Several studies have also found that children and adolescents are more likely to be developing ARFID (5, 6), which may be due to the fact that this period is crucial for growth and development, with a higher demand for nutrition. However, during this stage, the independence of group selection is enhanced, and the awareness of healthy diet is insufficient, which easily leads to diet avoidance (7). Additionally, some studies have suggested that it is related to gastrointestinal peptides, such as cholecystokinin (CCK) (8), orexigenic ghrelin, and anorexigenic peptide (PYY). Ghrelin is a peptide produced in the stomach, mainly secreted by P/D1 cells in the fundus of the stomach and pancreatic ϵ cells. It is responsible for regulating appetite, eating, and body composition, affecting adaptation and learning ability. PYY is an intestinal hormone that controls appetite. Studies have shown that ghrelin and PYY levels in patients with ARFID are higher than those in healthy controls (HC) and are also different from those in patients with anorexia (AN), indicating that these two hormones may be pathogenic factors for ARFID (9). In addition, ARFID is often accompanied by mental illness, such as feeding problems caused by insufficient care, eating disorders caused by anxiety, rigid eating behavior in autism spectrum disorders, and loss of appetite caused by disease stress. These mental factors play a key role in the pathogenesis of ARFID and increase the difficulty of clinical diagnosis.

As a newly included disease in diagnostic manuals, ARFID is currently understudied, and its symptoms can easily be confused with those of other disorders. The lack of objective biological markers makes it prone to clinical misdiagnosis or underdiagnosis, which can affect treatment and the health of patients. There are fewer epidemiological studies and diagnostic and therapeutic studies on this disease. International studies have also focused on the occurrence of the disease in children. This report presents a case of an adult male with ARFID and analyzes the patient’s medical history and treatment process. The aim is to provide clinical experience and a reference for early identification and correct treatment, hoping to improve clinical recognition and management of ARFID in adults.

## 2 Case description

A 57-year-old male was admitted to the Neurology Department’s Psychosomatic Sleep Ward of our hospital with the chief complaint of “decreased appetite for 7 months, aggravated with mental depression for 2 months”. Seven months before admission, the patient experienced a decrease in appetite without any obvious triggers, accompanied by brain buzzing, tinnitus, and hypersensitivity to sound, finding the environment noisy and intolerable. He sought treatment multiple times at the otolaryngology and neurology departments and underwent various examinations, but no significant abnormalities were found. The symptoms persisted without relief, affecting his sleep. He had previously taken Zopiclone and Dexzopiclone to aid sleep, but the effect was poor. At the same time, he experienced mood irritability; due to the fear of noise stimulation, he avoided contact with the outside world and dared not seek medical treatment outside. His family and household appliances tried to minimize noise to reduce stimulation for the patient. He had been diagnosed with generalized anxiety disorder but did not take anti-anxiety medication regularly.

Two months before admission, the patient experienced a gastric injury after eating corn and became afraid of suffering another gastric injury, leading to a reluctance to eat. This was accompanied by heart palpitations and chest tightness. He would worry that each mouthful of food could cause damage, so he mainly consumed only a small amount of congee. Twenty days before admission, his symptoms worsened, and he dared not eat anything, surviving only on water to alleviate hunger. His mental state deteriorated, resulting in difficulty falling asleep, frequent waking during the night, and difficulty falling back asleep after waking. He had constipation and normal urination, but his physical condition was significantly weakened, with a noticeable weight loss of 20 kg. His daily life was severely impacted, prompting him to seek treatment at our department. His BMI dropped to 16.7 kg/m². He denied having any negative thoughts or behaviors.

The patient had a history of good health, with no history of hepatitis, tuberculosis, malaria, hypertension, heart disease, diabetes, cerebrovascular disease, or psychiatric disorders. He also denied any gastrointestinal issues. There was no history of food or drug allergies. He had no history of smoking or alcohol consumption. On physical examination, his vital signs were as follows: body temperature of 36.5°C, heart rate of 80 beats per minute, respiratory rate of 20 breaths per minute, and blood pressure of 125/80 mmHg. His weight was 50 kg, and his height was 173 cm. The mental status examination revealed a depressed mood and reasonable responses to questions. He appeared anxious but had a neat appearance, normal thought processes, and no hallucinations or delusions were detected.

A comprehensive set of auxiliary tests was conducted, including a complete blood count, coagulation profile, D-dimer, FDP, myocardial markers, BNP, thyroid hormones, rheumatism indicators (anti-hemolytic streptococcus, rheumatoid factor, C-reactive protein), immunoglobulins (A, G, M, complement C3, C4), antinuclear antibody and titration tests (ANA), antinuclear antibody spectrum, anti-double-stranded DNA antibody, HIV, syphilis, hepatitis B, glycosylated hemoglobin, urine routine examination, and interleukin 6 (IL-6). No abnormalities were found. However, electrolyte tests showed a sodium ion level of 134 mmol/L, indicating hyponatremia.

**Figure 1:**
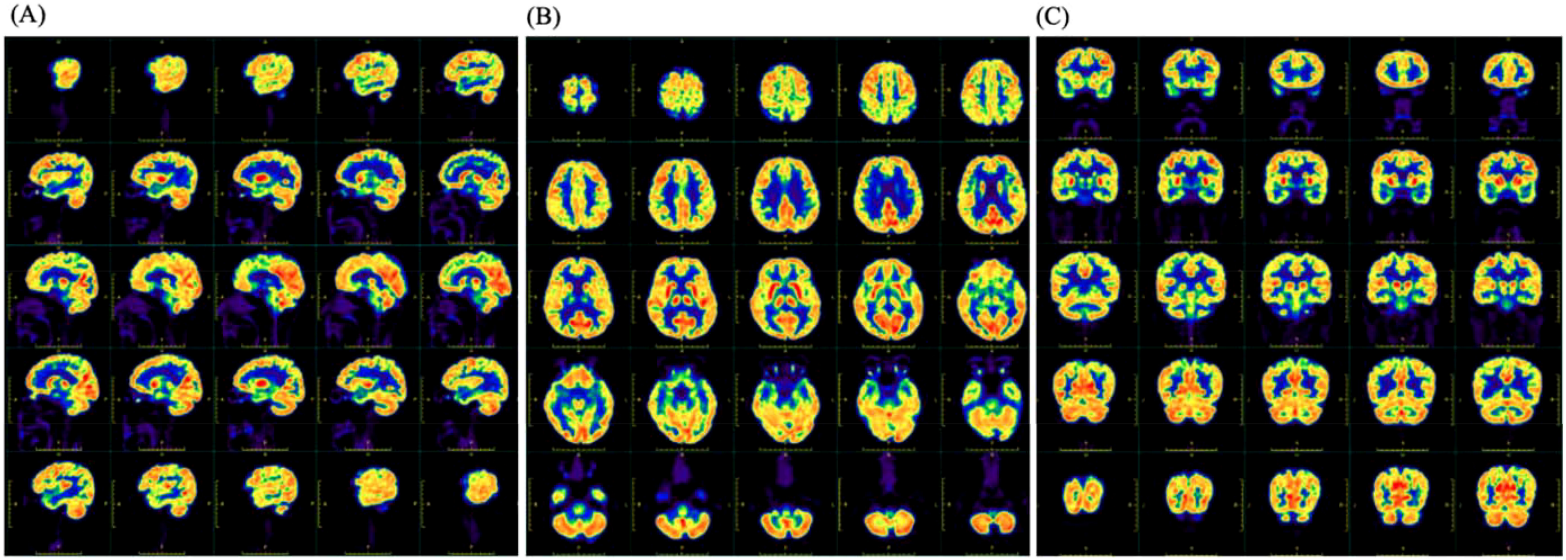
The image of ^18^F-FDG PET showed slightly decreased metabolism in the right parietal lobe, bilateral parts of the frontal lobe, left anterior cingulate gyrus, and right parietotemporal junction areas. **(A)** The sagittal position. **(B)** The transverse position. **(C)** The coronal position.

**Figure 2:**
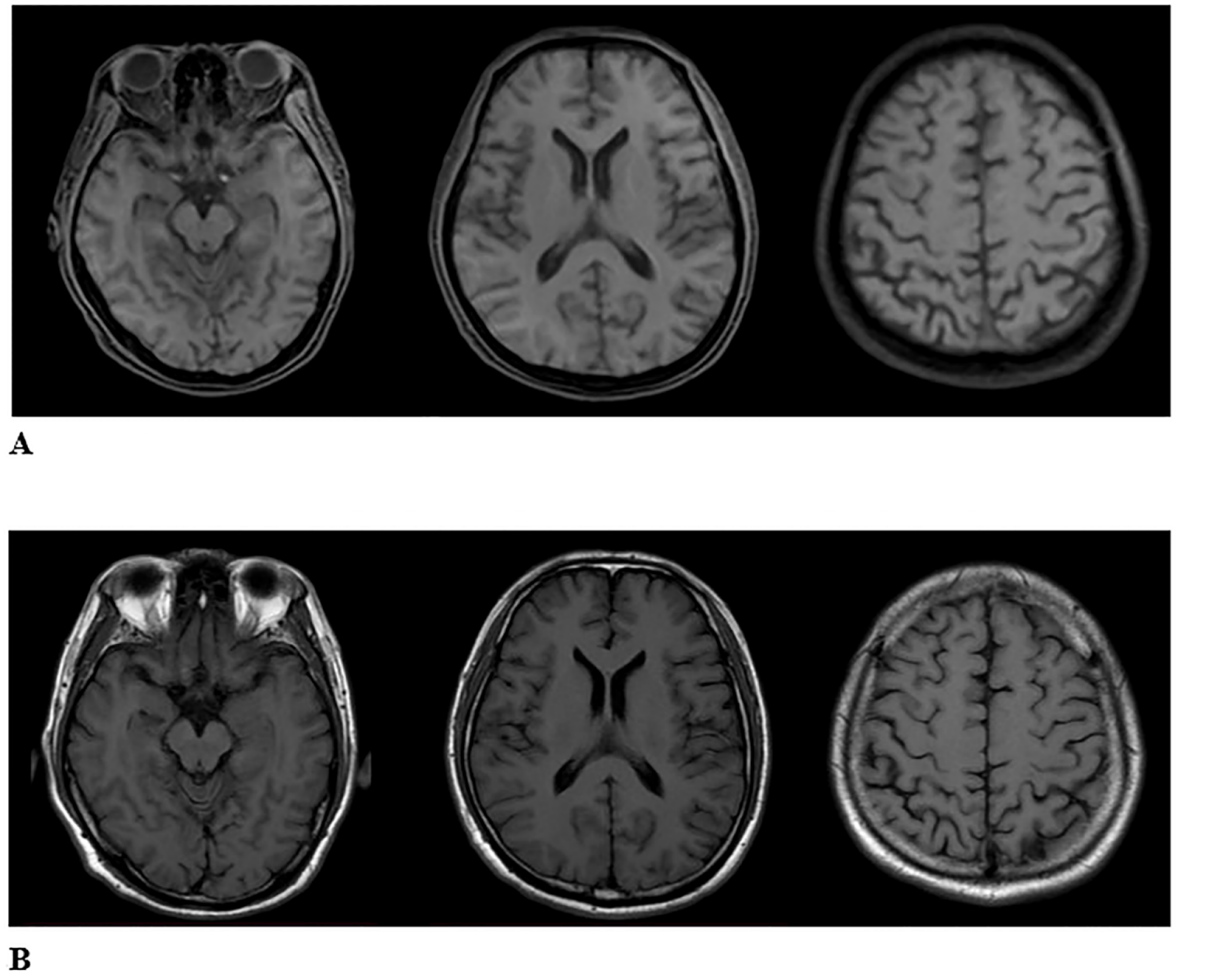
**(A)** The second image of MRI T1WI in ARFID state showed the bilateral frontal lobes and the right partial parietal lobe were slightly atrophied. **(B)** The first image of MRI T1WI in healthy state. There is no abnormality.

The Mini-mental State Examination (MMSE) got 27 points, and the Montreal Cognitive Assessment (MOCA) got 21 points. Cardiac electrocardiogram: sinus rhythm; cardiac axis is not perpendicular; ST-T changes. Abdominal ultrasound and urological ultrasound: enlarged gallbladder; thickened and hairy gallbladder wall; cholestasis; enlarged prostate with calcification; liver, spleen, pancreas, both kidneys, both ureters, and bladder did not show any obvious abnormality. Further detailed examination of the abdominal CT with enhancement showed: 1. low density shadow of the liver clavicular ligament side; consider the possibility of the clavical ligament side. 2. Cholestasis of the gallbladder. 3. Bipolar cystitis. Cardiac ultrasound showed that there was no obvious abnormality in the intracardiac structure, and the left ventricular systolic function was normal. Cervical vascular ultrasound and vertebral-basilar artery ultrasound showed plaque formation at the bifurcation of bilateral common carotid arteries. The bilateral vertebral artery and basilar artery blood flow velocity did not show an obvious abnormality. Color Doppler ultrasound of extremity vessels: femoral and popliteal veins of both lower limbs showed no abnormality. PET/MRI showed slightly decreased FDG (fluorodeoxyglucose) metabolism in the right partial parietal lobe, bilateral partial frontal lobes, left anterior cingulate gyrus, and right parietal-temporal joint region (**Figure 1**). The bilateral frontal lobes and the right partial parietal lobe were slightly atrophied in MRI (**Figure 2A**). On history review, the patient had a head MRI+MRA 7 months ago in his health state, which showed no abnormality (**Figure 2B**).

A psychological assessment was conducted, and during the diagnostic interview, the patient scored 40 on the Hamilton Anxiety Rating Scale, indicating severe anxiety, and the patient scored 33 on the Hamilton Depression Rating Scale, indicating severe depression. Organic causes for the eating disorder were ruled out. A gastric injury two months prior may have triggered an excessive focus on the gastrointestinal tract, leading to fear of gastrointestinal symptoms and a history of avoidance behaviors related to various health concerns, such as avoiding eating due to worries about the gastrointestinal tract. It needs to be emphasized that the patient did not have concerns about body image or an excessive focus on weight. He expressed a willingness to receive treatment and a desire to regain a normal weight. According to the diagnostic criteria of DSM-5, the patient meets the diagnostic criteria for ARFID, generalized anxiety disorder, and depressive episodes.

The patient’s treatment involved guidance from a nutritionist. On the first day of admission, nutritional support treatment consisted of a fat emulsion containing amino acid-17 glucose injections (1440 mL), injectable multivitamins (1co), and multiple trace element injections (6 mL). On the second day of admission, a compound sodium chloride injection of 500 mL was administered, along with enteral nutrition preparations: glucose sodium chloride injection of 500 mL, potassium chloride 15 mL, vitamin B6 0.1 g, and vitamin C 2 g. On the third to fifth days of admission, the patient received a glucose sodium chloride injection of 500 mL, potassium chloride of 15 mL, vitamin B6 of 0.1 g, and vitamin C of 2 g.

Taking into account the patient’s older age and the presence of severe anxiety disorder, the treatment was primarily medication-based. On the first day of admission, the patient was started on Olanzapine 2.5 mg at night (Qn), Estazolam 0.5 mg once a day (Qd), and Escitalopram Oxalate 10 mg Qd. On the 13th day of admission, the dose of Escitalopram Oxalate was increased to 15 mg Qd, and Fluoxetine Hydrobromide 5 mg (taken with dinner) was added orally. On the 15th day of admission, the dose of fluoxetine hydrobromide was increased to 10 mg (taken with dinner) orally. The patient’s condition showed improvement over time with the treatment regimen. By the 18th day after admission, the patient’s appetite had improved, as had their anxiety symptoms and sleep quality. Their weight increased to 54 kg, and their BMI rose to 18.04 kg/m².

Following discharge, the patient continued with regular follow-up appointments (**Table 1**). On November 17, 2023, during a follow-up visit, the patient reported occasional headaches, good night sleep, increased appetite, and a good diet. The medication regimen at that time included fluoxetine hydrobromide 10 mg Qn, Escitalopram Oxalate 15 mg Qd, and Olanzapine 1.25 mg Qn. By December 13, 2023, the patient had experienced anxiety and a sense of oppression, along with significant tinnitus, leading to an increase in the dose of Escitalopram Oxalate to 20 mg Qd. The other medications remained the same. On January 15, 2024, the patient showed good improvement in symptoms but had concerns about elevated blood sugar levels. The medication regimen was adjusted to Fluoxetine Hydrobromide 10 mg Qn, Escitalopram Oxalate 20 mg Qd, and an eighth of a tablet of Olanzapine Qn. By March 18, 2024, the patient continued to show good improvement, and the medication was stable at Fluoxetine Hydrobromide 10 mg Qn and Escitalopram Oxalate 20 mg Qd. On April 22, 2024, the patient’s condition was still improving well, and the medication was adjusted to Fluoxetine Hydrobromide 10 mg Qn and Escitalopram Oxalate 15 mg Qd. Then, on May 13, 2024, the patient was still doing well, and the medication was further adjusted to Fluoxetine Hydrobromide 5 mg Qn and Escitalopram Oxalate 15 mg Qd. Finally, on Nov 18, 2024, the patient ‘s appetite returned to normal, and the mood improved, and the Escitalopram Oxalate was reduced to 10 mg Qd, and Fluoxetine Hydrobromide was still 5 mg Qn.

**Table 1:** Drug dosage for patient follow-up after discharge.

Follow up	2023.11.17	2023.12.13	2024.01.15	2024.03.18	2024.04.22	2024.05.13	2024.11.18
Escitalopram Oxalate	15mgQD	20mgQD	20mgQD	20mgQD	15mgQD	15mgQD	10mgQD
Fluoxetine Hydrobromide	10mgQN	10mgQN	10mgQN	10mgQN	10mgQN	5mgQN	5mgQN
Olanzapine	1.25mgQN	1.25mgQN	0.625mgQN	0.625mgQN	-	-	–

## 3 Diagnostic

In this case, the patient was unable to eat, had no weight or size requirements, and could not be explained by physical illness or other mental disorders. The patient’s fear of eating, which stems from a gastric injury after eating corn, leads to a restriction of intake to only a small amount of congee, accompanied by stomach pain and discomfort. This fear of damaging the stomach through eating, combined with the stereotype of experiencing stomach pain after meals, has resulted in an excessive awakening and paranoid cognition regarding the symptoms themselves. Additionally, the patient’s anxiety drives a cognitive pattern of attributing benign sensations to catastrophic outcomes.

## 4 Discussion

ARFID is a newly recognized condition in the DSM-5. According to the DSM-5 criteria for ARFID, the diagnostic features involve avoidance or restriction of food intake, with the requirement that at least one of the following be present: 1) significant weight loss; 2) significant nutritional deficiencies; 3) dependence on enteral feeding or oral nutritional supplements; 4) significant interference with psychological or social functioning (1). The avoidance or restriction of food intake is not due to a lack of availability of certain foods or related to cultural practices around eating. It is also not explained by other eating disorders or by concerns about body shape or weight. To rule out other potential causes, a thorough workup was conducted, including tests for hepatitis viruses, syphilis, HIV, tumor markers, and imaging studies of the head and abdomen, all of which yielded normal results. There was no evidence of concurrent somatic illnesses or other psychiatric disorders that could account for the eating pattern. Given the absence of concerns about weight or body shape and the significant impact on the patient’s physical and mental health due to the avoidance or restriction of food, the final diagnosis considered was ARFID.

Currently, there are several assessment tools available for evaluating ARFID: ① EDY-Q (Eating Disturbances in Youth-Questionnaire): This tool is designed for use with patients aged 8 to 13 years. It is a self-report questionnaire that assesses various aspects of eating disorders in children and adolescents (10). ② NIAS (Nine Item Avoidant/Restrictive Food Intake Disorder Screen): This screening tool is intended for use with individuals aged 18 to 65 years. It consists of nine items that are used to identify potential cases of ARFID (11). ③ ARFID-Q-PR (Avoidant/Restrictive Food Intake Disorder Questionnaire Parents Report): This version of the ARFID questionnaire is completed by parents and is suitable for use with children (12). These tools are designed to assist healthcare professionals in the diagnosis and assessment of ARFID by providing structured evaluations of the symptoms and behaviors associated with the disorder. In addition to self-report questionnaires, there are semi-structured interviews that can be used to assess ARFID. These include: ① PARDI (Pica, Avoidant/Restrictive Food Intake Disorder, and Rumination Disorder Interview) (13): This is a semi-structured interview that can be used to assess children and adults for the presence and severity of ARFID (14). It provides three dimensions for rating ARFID cases: eating diversity, food neophobia tendency, and psychosocial impairment. PARDI also evaluates the core constructs of the psychopathology of ARFID, such as dietary variety, fear of new foods, and psychological and social distress. It is suitable for use with children, adolescents, and adults. ② EDA-5 (Eating Disorders Assessment for DSM-5): This is a brief, online semi-structured clinical interview designed to assess for eating disorders (15). While it has extensive validity data for other eating disorders (such as anorexia nervosa and bulimia nervosa), there is less data available for ARFID. ③ SCID-5 (Structured Clinical Interview for DSM-5): This is another semi-structured interview used to provide DSM-5 diagnoses (16). Its module for eating disorders assesses all diagnostic criteria and age of onset for feeding and eating disorders. Semi-structured interviews serve as essential tools for clinicians, offering insights into patients’ eating habits and psychological aspects, crucial for precise ARFID diagnosis and treatment.

**Figure 3:**
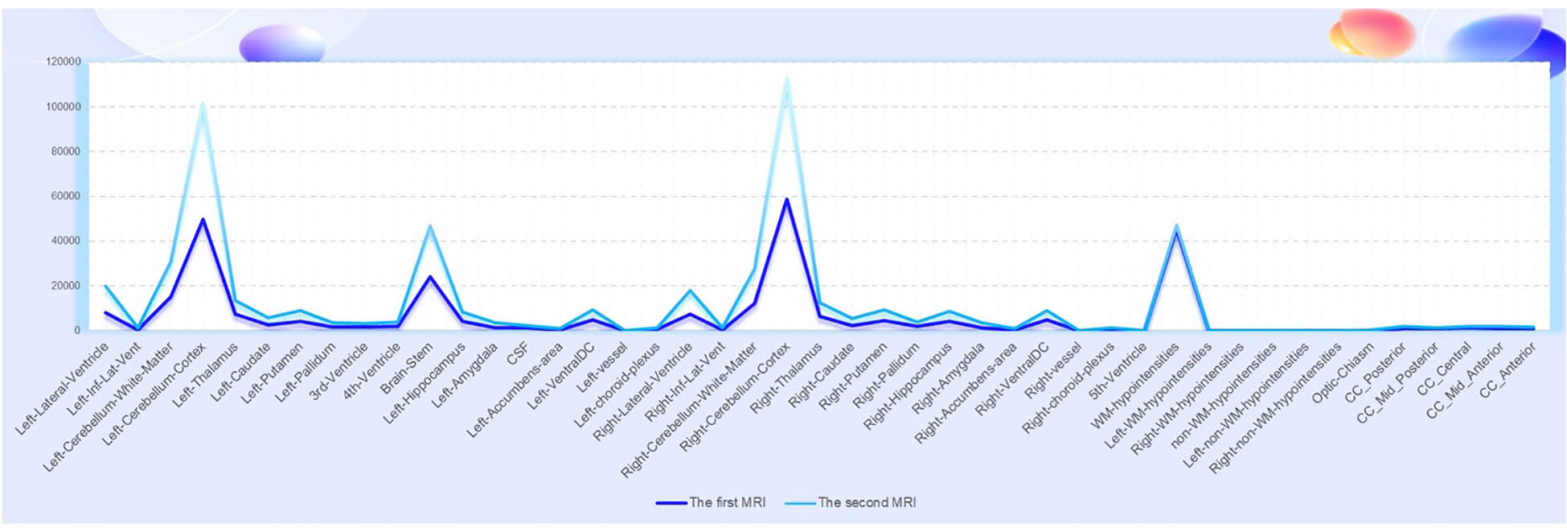
The comparison of the imaging findings between the first MRI (healthy state) and the second MRI (ARFID state) revealed an increase in the volume of some brain regions and nuclei.

Currently, the diagnosis of ARFID relies on clinical scales and semi-structured interviews, with a lack of objective biological markers or radiological examinations to distinguish it from other eating disorders and psychiatric conditions. Head imaging studies specifically for ARFID are rare, but in this particular case, a head MRI was conducted on the patient during their healthy state before the onset of ARFID, and an 18F-FDG PET/MRI scan was performed after the onset of the disorder. Despite the limited data from a single typical case, the comparison of the imaging findings between the healthy state and the state of ARFID revealed an increase in the volume of some brain regions and nuclei (**Figure 3**), which may be related to compensatory changes in functional areas. Research on imaging in other eating disorders, such as anorexia nervosa, has primarily focused on brain regions like the insula, orbitofrontal cortex, ventral striatum, and hypothalamus (17, 18). These studies have proposed theories related to networks involved in executive function, reward processing, and perception (19, 20). While ARFID-specific imaging studies are lacking, insights from other eating disorders can inform the understanding of potential neurobiological mechanisms underlying ARFID and its relationship to other psychiatric conditions. Further research is needed to investigate the neuroimaging characteristics of ARFID and to explore potential biomarkers that could aid in the diagnosis and understanding of the neurobiology of this disorder (21).

Furthermore, in the context of imaging for ARFID, it is important to use imaging as a method to exclude secondary causes of eating disorders, such as tumors or other brain-related organic factors (22). This is particularly relevant for diagnosing ARFID and other eating disorders, as it is crucial to rule out such secondary causes. There is hope for future studies to include larger retrospective or prospective cohorts, which could contribute to the development of imaging biomarkers for ARFID. With the increasing application of PET in neuropsychiatric disorders, it is anticipated that future research will provide insights into the role of PET imaging in ARFID, although there is currently a lack of large-scale PET studies in this area. In the case presented, FDG metabolism imaging revealed slightly reduced glucose metabolism in the right parietal lobe, bilateral parts of the frontal lobe, left anterior cingulate gyrus, and right parietotemporal junction areas. This finding suggests that further research is needed to understand the neurobiological underpinnings of ARFID and its relationship to glucose metabolism in specific brain regions. The reported case study is expected to provide new directions for future research on ARFID and its potential imaging biomarkers.

ARFID is often accompanied by nutritional deficiency, which can lead to serious complications. Treatment includes intravenous nutrition, oral nutrition powder and nasal feeding. Lack of vitamin B12, zinc, iron, calcium, folic acid or protein, etc., can lead to a variety of symptoms (23), such as fatigue, poor growth, skin problems, anemia (24), decreased immunity, etc. Nutritional disorders caused by ARFID may lead to Wernicke encephalopathy (25–27), and early identification and management should be emphasized (6). In addition to nutritional therapy, treatment also includes cognitive behavioral therapy and drug therapy. The current research focuses on children ‘s psychological cognitive behavioral therapy, such as parental psychological intervention. Studies have found that psychological intervention helps to establish a correct concept of eating (25). The main treatment methods are Cognitive Behavioral Therapy (CBT) (28) and Family Based Treatment (FBT) (29, 30), and there is a special Cognitive Behavioral Therapy for ARFID (CBT-AR) treatment, which is divided into four stages for ARFID characteristics (31, 32). Studies have shown that CBT-AR is effective for children, adolescents and adults (31, 33–35). A study has shown that exposure-based CBT treatment can improve dietary diversity and psychosocial function, but some patients still have residual symptoms, and further research is needed to verify the long-term effect of CBT (36).

**Figure 4:**
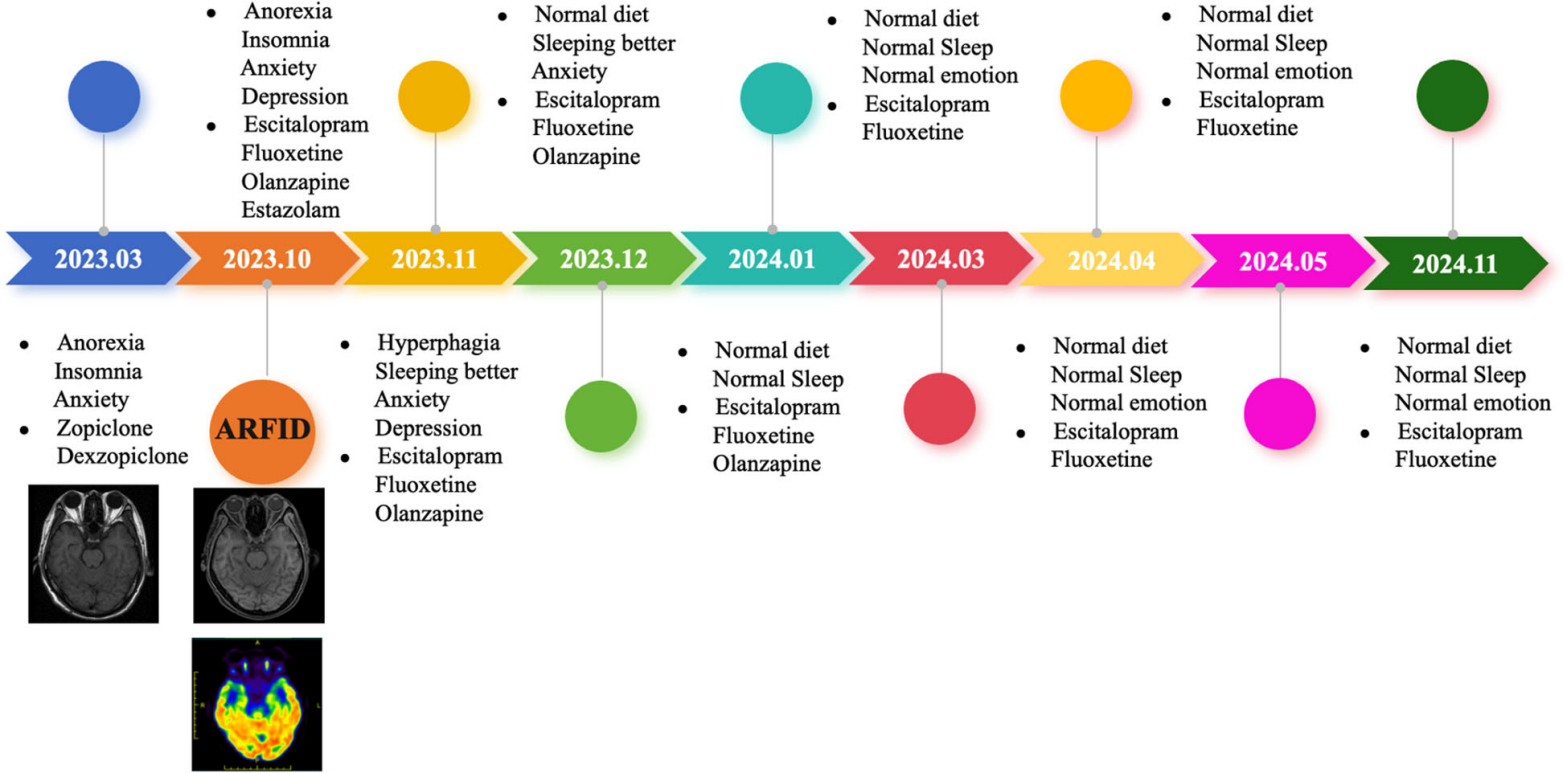
Timeline of case.

Despite the wide clinical application of cognitive-behavioral therapy, it is particularly effective for children and young patients. CBT has a long treatment cycle and requires high patient compliance. It becomes more challenging to change ingrained thoughts in a short period of time as patients age. Therefore, for this patient, in addition to symptomatic nutritional support therapy, we provided medication treatment, which is effective in the short term, improves appetite, and enhances the patient’s eating level. Moreover, it can also improve the patient’s co-occurring anxiety and depression symptoms (**Figure 4**). It has been reported that low doses of olanzapine, an atypical antipsychotic, can assist in promoting eating, increasing weight, and improving symptoms of anxiety and depression, as well as relieving cognitive impairment (37). In addition to olanzapine, a class of atypical antipsychotics, the SSRI (selective serotonin reuptake inhibitor) antidepressants seem to have a certain effect on the treatment of ARFID. Case reports have shown that escitalopram and fluoxetine have a therapeutic effect on ARFID (27, 38–40), but current research is still in the preliminary exploration stage. In terms of medication treatment for ARFID, we have initially tried low doses of olanzapine in combination with escitalopram oxalate and fluoxetine hydrobromide, which has achieved some therapeutic effects. However, this represents only individual cases, and medication treatment still requires large-scale, randomized, double-blind studies to fully validate its efficacy.

## 5 Conclusion

ARFID, as a disease newly included in the diagnostic criteria, is still not well understood, and its recognition by clinicians is challenging. In addition, it is essential to clarify epidemiological data, including its incidence, risk factors, pathogenesis, clinical characteristics, and the findings of supportive diagnostic tests. It provides help for clinicians to accurately identify the disease, so as to treat the disease in time. Generally, patients with this kind of disease will be tossed in various departments of the hospital before they are correctly treated in the department of psychiatry, and do a lot of examinations repeatedly, which wastes a lot of medical resources, delays the diagnosis and treatment of the disease, and seriously endangers the life of the patients. Therefore, for clinically relevant departments, we should understand the disease and identify it in time, so that patients can avoid detours. In addition, the report of the disease will help clinicians to improve the vigilance of the disease, combined with neuroimaging and other disciplines for comprehensive diagnosis and treatment, and provide reference for future research on adult ARFID.

## Data Availability

The original contributions presented in the study are included in the article/supplementary material. Further inquiries can be directed to the corresponding authors.
